# Examining Global Crises: Extracting Insights From the COVID-19 Pandemic and Natural Disasters to Develop a Robust Emergency Diabetic Retinopathy Strategy for Puerto Rico

**DOI:** 10.7759/cureus.47070

**Published:** 2023-10-15

**Authors:** Gabriel Guardiola Dávila, José J López-Fontanet, Fabiola Ramos, Michael A Acevedo Monsanto

**Affiliations:** 1 Department of Ophthalmology, Medical Sciences Campus, University of Puerto Rico, San Juan, PRI; 2 Department of Internal Medicine, Mayo Clinic, Jacksonville, USA

**Keywords:** covid-19, puerto rico, natural disaster, ophthalmology, diabetic retinopathy

## Abstract

In this critical analysis, we investigate the profound impact of natural disasters and pandemics on the care and adherence to treating diabetic retinopathy, a severe complication of diabetes requiring continuous monitoring and treatment to prevent vision loss. Our study also sheds light on the social and economic context of Puerto Rico, emphasizing recent emergency events that have exacerbated existing public health challenges. Through a comprehensive review of relevant literature from PubMed, Google Scholar, and the George Washington University Himmelfarb Health Sciences Library database, we identified 31 pertinent articles out of 45 evaluated, focusing on the effects of these crises on healthcare delivery, diabetic retinopathy screening, and treatment. The evidence strongly indicates that during such emergencies, barriers to healthcare escalate, leading to significant treatment delays and a reduction in diabetic retinopathy screening and diagnosis, ultimately resulting in deteriorated visual outcomes. Thus, our review underscores the urgent need for the development of effective emergency plans tailored specifically to diabetic retinopathy, particularly in Puerto Rico, where diabetes prevalence and its complications are notably higher. Such plans should not only incorporate established emergency measures but also harness emerging technological advances in the field of ophthalmology to ensure optimal preparedness for future pandemics and natural disasters.

## Introduction and background

Diabetes, characterized by elevated blood glucose levels, poses a global health challenge with both macrovascular and microvascular complications. The incidence of diabetes is rising worldwide, affecting vulnerable populations, especially in disaster-prone regions [1]. The International Diabetes Federation reported an alarming prevalence of diabetes, projecting a further increase by 2030 [2]. Notably, Hispanics, particularly in Puerto Rico, face a higher burden of diabetes, contributing to increased complications and mortality [3,4]. Among these complications, diabetic retinopathy (DR), a microvascular disorder of the retina, stands out as a leading cause of preventable blindness in adults [5,6].

The prevalence of DR is escalating, especially in regions like North America and the Caribbean, with Puerto Rico experiencing a notable burden [7,8]. This burden is compounded by the recurrent hurricane seasons [4], which underscores the urgent need for effective disaster preparedness strategies. In understanding the impact of disasters on DR, the nature of catastrophes, community resilience, and healthcare resources play pivotal roles [9]. Disasters disrupt access to essential resources like healthy diets and medications, potentially worsening diabetes and its complications [10]. Consequently, disaster-related disparities could exacerbate the challenges faced by diabetic patients, particularly those with retinopathy [10].

Prior research underscores the exacerbation of diabetes and its complications in disaster settings [11,12]. Diabetic patients often experience increased hospitalizations, acute complications, and disruptions in blood sugar control [11,12]. Even months after disasters like earthquakes and hurricanes, patients exhibit elevated hemoglobin A1c levels, highlighting the long-term impact on glycemic control [10,12]. Access to medications becomes a critical concern during emergencies, as disruptions can hinder proper disease management [10]. Given these challenges, it is evident that effective strategies are necessary to mitigate the impact of disasters on DR.

DR management revolves around prevention and progression control [5]. While blood glucose control helps prevent its onset, managing established retinopathy demands intensive interventions [5,13]. These interventions range from regular ophthalmological visits for early detection to advanced procedures like laser therapy and intravitreal injections [5,13]. Emergencies like natural disasters and pandemics pose a unique threat to high-risk individuals already undergoing DR treatment [5]. This highlights the urgency of formulating contingency plans to safeguard patients and minimize irreversible consequences.

This critical analysis aims to elucidate the influence of natural disasters and pandemics on DR care and adherence to treatment. Moreover, it sheds light on Puerto Rico's recent public health challenges, magnified by its susceptibility to disasters [4]. Through a comprehensive examination of the existing literature, this review emphasizes the need for tailored preparedness measures targeting DR.

## Review

Materials & methods

A comprehensive literature review was conducted to identify relevant publications on managing DR during pandemics or natural disasters worldwide. The study also aimed to identify recent public health challenges in Puerto Rico that may have affected the care of DR on the island during and after Hurricane Irma, Hurricane Maria, the 2019-2020 earthquakes, and the COVID-19 pandemic. Additionally, the research sought to assess the presence of diabetes emergency preparation procedures on the island. Furthermore, given the ongoing COVID-19 pandemic, the literature review explored suggested methods and new technological breakthroughs for managing DR.

Literature searches were conducted using PubMed, Google Scholar, and the George Washington University Himmelfarb Health Sciences Library database. The search queries focused on terms related to DR, as it is the primary disease of interest in this critical analysis. Similarly, words related to disaster preparedness were included. The search terms used had "diabetic retinopathy" ("retinopatia diabética"), "retinopathy" ("retinopatia"), "retina," "diabetes," "diabetic eye disease," "diabetes mellitus," and "diabetics" ("diabéticos"), along with "disaster preparedness," "emergency preparedness," "natural disasters" ("desastres naturales"), "hurricanes" ("huracanes"), "Hurricane Maria" ("Huracán Maria"), "Hurricane Irma" ("Huracán Irma"), "earthquakes" ("terremotos"), "pandemic" ("pandemia"), "covid," "covid-19," and "coronavirus." Additional terms such as "prevalence" ("prevalencia"), "Puerto Rico," "intravitreal injections," "anti-vascular endothelial growth factor," and "anti-VEGF" were used to achieve specific aims.

Appendix A provides a list of combinations of the search terms mentioned above. Studies included in the literature review were limited to those written in English or Spanish, available as free full-text, and published from 2017 onwards.

Results

**Figure 1:**
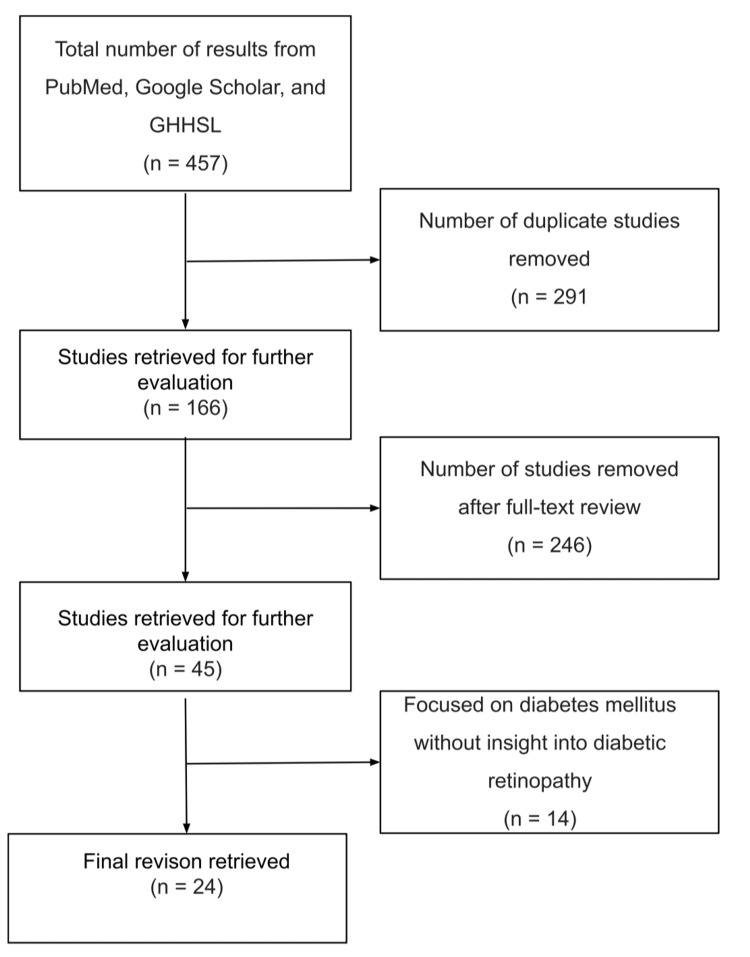
Flowchart illustrating the literature inclusion and exclusion criteria for article selection GHHSL: George Washington University Himmelfarb Health Sciences Library.

This study conducted an extensive literature review spanning the period from 2017 to 2022, with the aim of delving into the intricate dynamics between natural disasters, pandemics, and the provision of care for DR. A meticulous analysis of 24 relevant articles provided valuable insights into the challenges faced by DR patients during times of adversity, as depicted in Figure 1. The distribution of study types across the reviewed literature is succinctly captured in Table 1.

**Table 1:** Distribution of study types in the reviewed literature

Study type	Count	Percentage
Literature reviews	11	46%
Retrospective studies	8	33%
Prospective studies	5	21%

Remarkably, as highlighted in Table 2, a significant portion of the reviewed studies evaluated the implications of delays in administering anti-vascular endothelial growth factor (anti-VEGF) treatment to patients with DR during the COVID-19 pandemic [14-21]. Noteworthy advances in DR screening and diagnostic technologies were a recurrent theme across multiple studies [22-28]. A subset of articles scrutinized the impact of COVID-19 lockdowns on DR, with contributions from various nations, including Greece [29], Jordan [30], the United States [14], and India [31]. Furthermore, diverse perspectives emerged as the literature explored alternative strategies for fundoscopic imaging [32-35] and DR treatment and barriers [36,37] that hinder patients' adherence to follow-up care.

**Table 2:** Division of reviewed studies by main topics * Articles that are repeated in more than one category. DR: diabetic retinopathy; VEGF: vascular endothelial growth factor.

Topic	Studies	Count
Anti-VEGF treatment delays during COVID-19	Douglas et al. (2022)* [14]; El Hamichi et al. (2019)* [15]; Moussa et al. (2021) [16]; Naravane et al. (2021) [17]; Rush & Rush (2021) [18]; Wasser et al. (2020) [19]; Weng et al. (2021) [20]; Ahmed & Liu (2021) [21]	8
Digital advances in DR screening and diagnosis	Benet & Pellicer-Valero (2022) [22]; Galiero et al. (2020) [23]; Hristova et al. (2021) [24]; Li et al. (2021) [25]; Nikolaidou & Tsaousis (2021) [26]; Raparia & Husain (2021) [27]; Sommer & Blumenthal (2020) [28]	7
Effects of COVID-19 lockdown on DR	Al-Dwairi et al. (2021) (Jordan) [29]; Chatziralli et al. (2021) (Greece) [30]; Douglas et al. (2022)* (United States) [14]; Das et al. (2021) (India) [31]	4
Alternative strategies for fundoscopic imaging	Chen et al. (2021) [32]; Kato et al. (2021) [33]; Kumari et al. (2022) [34]; Queiroz et al. (2020) [35]	4
Barriers to follow-up care and treatment compliance	Lindeke-Myers et al. (2021) [36]; Shields et al. (2021) [37]	2
Disaster preparedness strategies for DR	El Hamichi et al. (2019)* [15]	1

Intriguingly, the literature appeared to have a significant gap in the specific domain of disaster preparedness strategies tailored to DR during natural disasters [15], a point highlighted in Table 2. Only one article was identified to address this crucial aspect, emphasizing a potential avenue for future research.

The synthesis of the literature convincingly underscores the susceptibility of DR care to the disruptions posed by natural disasters and pandemics. The aftermath of events like hurricanes and the ongoing COVID-19 pandemic has underscored challenges in timely care provision, leading to delays in vital interventions such as anti-VEGF injections and DR screenings. These delays can potentially worsen the prognosis of DR and heighten the risk of vision loss. Of significant note, the region of Puerto Rico, having endured multiple natural disasters in the past decade alongside the ongoing pandemic, is a vivid example of the disproportionate impact on individuals with chronic illnesses. These crises have laid bare shortcomings in the healthcare system and accentuated the pressing need for robust contingency plans to mitigate considerable health and visual consequences. It is imperative that such plans encompass both established emergency protocols and the integration of emerging technological advancements within the field of ophthalmology.

Impact of Pandemics and Natural Disasters on Diabetic Retinopathy: Challenges and Preparedness Strategies

The ramifications of pandemics and natural disasters on DR care have garnered significant attention within the medical community. This section synthesizes key findings from the literature, shedding light on the barriers encountered by DR patients during these emergency situations and proposing effective preparedness strategies to mitigate their impact.

Barriers to care during the COVID-19 pandemic: The COVID-19 pandemic highlighted several barriers that disrupted the continuity of DR care. As depicted in Table 3, these barriers included decreased clinic visits and patient volumes, often driven by patient fears, extended wait times, and financial concerns. Consequently, follow-up appointments and essential treatments were delayed, disproportionately affecting high-risk patients who were more susceptible to loss of follow-up due to COVID-19 concerns. Moreover, delays in anti-VEGF therapy led to worsened anatomical outcomes, further accentuating the negative impact. Lockdown measures resulted in reduced DR screenings, posing significant risks of undiagnosed cases and delayed treatment. These findings emphasize the critical nature of these barriers in hampering effective DR management during pandemic scenarios.

**Table 3:** Impact of barriers during the COVID-19 pandemic on DR care DR: diabetic retinopathy; VEGF: vascular endothelial growth factor.

Barriers to care	Effects on DR care
Decreased clinic visits and patient volumes	Reduced access to regular screenings and treatment
Patient fears and extended wait times	Delayed follow-up appointments and treatment
Financial concerns	Limited access to necessary medications and treatments
High-risk patients lost to follow-up	Increased risk of irreversible visual impairments
Delays in anti-VEGF therapy	Worsened outcomes
Reduced diabetic retinopathy screening due to lockdowns	Risks of undiagnosed cases and delayed treatment

Impact of recent emergency situations in Puerto Rico: Existing healthcare challenges and socioeconomic factors amplified Puerto Rico's vulnerability to emergencies. Table 4 underscores the repercussions of recent emergency situations, including hurricanes, earthquakes, and the COVID-19 pandemic, on DR care. These emergencies disrupted healthcare infrastructure and strained healthcare resources and personnel, disproportionately affecting the diabetic population. Moreover, socioeconomic challenges, such as limited access to care for rural residents and the elderly, combined with the concentration of hospitals in metropolitan areas, further exacerbated the difficulties faced by vulnerable populations. These findings emphasize the intricate connection between natural disasters, healthcare infrastructure, and chronic disease management, advocating for a comprehensive emergency response plan.

**Table 4:** Impact of recent emergencies on DR care in Puerto Rico DR: diabetic retinopathy.

Emergency situations	Challenges faced in DR care
Hurricanes	Limited access to care due to infrastructure damage
Earthquakes	Disruption of healthcare facilities
COVID-19 pandemic	Strain on healthcare resources and personnel
Socioeconomic issues	Access barriers for rural residents and elderly
Concentration of hospitals	Overburdened metropolitan facilities

Proposed pandemic and disaster preparedness plan for DR: To address the challenges highlighted by the impact of emergencies on DR care, a multifaceted preparedness plan is proposed. Table 5 outlines these strategies, which collectively aim to enhance the resilience of DR care during crises. The plan includes the establishment of a diabetic retinopathy registry and surveillance system, allowing real-time estimation of disease prevalence to guide resource allocation. Identifying barriers and high-risk patients enables targeted interventions, thereby reducing delays in follow-up and treatment and preventing irreversible visual impairments. The concept of exemptions for high-risk patients from lockdown measures ensures the continuity of care while adhering to safety protocols. Integration of telemedicine and AI technologies enables remote consultations and diagnoses, minimizing exposure risks. Portable retinal imaging devices offer convenient screening opportunities, especially valuable during emergencies. Lastly, individualized emergency plans encompass actions such as medication stockpiling and alternative communication methods, ensuring uninterrupted DR care.

**Table 5:** Proposed pandemic and disaster preparedness plan for DR DR: diabetic retinopathy.

Strategies	Implementation and benefits
Diabetic retinopathy registry and surveillance system	Real-time data for resource allocation and identifying vulnerable areas.
Identify barriers and high-risk patients	Targeted interventions to prevent delays and visual impairments.
Exemptions for high-risk patients	Ensures continuous care during emergencies while maintaining safety.
Telemedicine and artificial intelligence integration	Remote consultation and diagnosis, reducing exposure risks.
Portable devices for retinal imaging	Convenient screening and remote consultation capabilities.
Individualized emergency plans	Uninterrupted care through stockpiling and alternative communication.

Discussion

The prevalence of DR, a significant microvascular complication of diabetes mellitus, presents a formidable challenge due to its potential to cause vision impairment and blindness among working-age individuals. This condition is chiefly characterized by diabetic macular edema (DME) and proliferative diabetic retinopathy (PDR), necessitating timely intervention and vigilant monitoring [30]. The conventional approach to managing DR involves periodic intravitreal anti-VEGF injections administered by certified ophthalmologists. However, unexpected events like natural disasters and pandemics can disrupt DR screening and treatment, negatively affecting visual outcomes and quality of life. A poignant example of this disruption is evident in the consequences of the COVID-19 pandemic. Concerns over infection risk, transportation limitations, healthcare accessibility, and increased expenses have led to delayed care and reduced adherence to anti-VEGF treatments [16,23,36,37]. The imposition of social distancing measures during the pandemic has further impeded office visits and the frequency of DR examinations [21].

To mitigate the repercussions of these care disruptions, ophthalmological institutions have outlined recommendations for managing intravitreal anti-VEGF injections during pandemics [30]. These guidelines underscore the need to minimize exposure for both patients and healthcare personnel, prioritize treatment for individuals at high risk of vision loss, and explore simplified anti-VEGF injection regimens [30]. Integrating telemedicine and digital innovations in ophthalmology has emerged as a promising solution to enhance disaster preparedness for DR care. Hence, it is crucial to incorporate the latest technological advancements into a comprehensive analysis of strategies for managing DR during emergencies.

Telemedicine has proven invaluable when conventional medical access is limited, as witnessed in situations like natural disasters and infectious disease outbreaks. The COVID-19 pandemic has expedited the adoption of telemedicine, enabling remote healthcare delivery through digital platforms. Ophthalmology has particularly benefited from this shift, leveraging advanced hardware, sophisticated software, and high-speed communication technologies to remotely diagnose and manage eye conditions [26,28]. The amalgamation of telemedicine and ophthalmology, known as teleophthalmology, holds transformative potential in care delivery, especially during crises. This approach has shown promise in managing retinal diseases like DR, age-related macular degeneration, and retinopathy of prematurity, alongside conditions like glaucoma and anterior segment diseases [26]. Integrating artificial intelligence (AI) algorithms with teleophthalmology can further enhance diagnostic accuracy and treatment outcomes, with AI assisting in interpreting retinal images, facilitating remote evaluation by ophthalmologists, or automated analysis by deep learning algorithms [22].

Teleophthalmology, mainly via portable devices, has the capacity to bridge accessibility gaps in remote and underserved communities. Research has demonstrated the feasibility of cost-effective DR screening tools utilizing smartphones and telemedicine, resulting in improved DR screening coverage in rural areas [15]. Furthermore, advancements in laser photocoagulation devices have enhanced precision and user-friendliness, making them well-suited for integration into telemedicine, thus enabling remote real-time consultation and expert care, which in turn improves therapeutic outcomes and reduces collateral retinal damage in the context of DR treatment [34]. While teleophthalmology does have limitations, its benefits are evident.

In addition to leveraging telemedicine, public health initiatives should prioritize infrastructure enhancements to bolster visual healthcare accessibility. Strengthening hospitals, medical facilities, and primary care clinics, ensuring consistent power supply and backup generators, improving provider communication, expanding the healthcare workforce, enhancing surveillance systems, and refining patient records are imperative. Special attention should be directed toward high-risk groups, such as elderly individuals with chronic conditions and immobile patients [38]. Expanding broadband internet access, particularly in rural regions, is vital for improved communication and healthcare access. Transportation alternatives must also be improved to facilitate patient mobility.

Community pharmacists play a pivotal role in disaster preparedness and response, offering access to medications and healthcare services, thereby mitigating barriers to care [39]. They can contribute significantly to patient care and drug/vaccine distribution during crises, reducing adverse health impacts. Similarly, community-based organizations (CBOs) play a crucial role in disaster management, particularly in aiding older and disabled individuals in accessing appropriate care. CBOs can complement governmental initiatives, as evidenced during the COVID-19 pandemic and post-Hurricane Maria recovery efforts [40].

National-level protocols should be established to ensure uninterrupted critical treatment schedules for DR patients. These protocols must recognize the debilitating nature of the disease and prioritize patients at high risk of DR-related blindness, exempting them from lockdown measures and ensuring their protection [29]. Similar to prioritizing cancer patients during emergencies, individuals with vision-threatening conditions like DR, requiring frequent monitoring and treatment, should receive priority attention. Research suggests that modifying anti-VEGF injection schedules and treatment frequencies during emergencies should be considered. For less severe DR cases, postponing anti-VEGF injections to prioritize more severe cases has been recommended [30]. Exploring approaches such as the treat-and-extend regimen, home screening via portable optical coherence tomography examinations, and newer long-acting anti-VEGF medications is imperative [29].

The COVID-19 pandemic has instigated transformative shifts in care delivery and spurred innovative standards in ophthalmic care. As the global population ages, the prevalence of DR and similar conditions will escalate, further straining healthcare services. In regions like Puerto Rico, where diabetes mellitus is highly prevalent, and healthcare infrastructure and resources might be less stable, swift integration of alternative care methods, such as telemedicine, is crucial to alleviate patient burden while ensuring essential disease management [25]. Telemedicine offers advantages in screening, monitoring, and triaging patients, enabling ophthalmologists to closely oversee patients remotely, with support from optometrists, ophthalmic technicians, and non-medical healthcare professionals for remote retinal image grading. Comprehensive research and careful implementation are imperative for effectively utilizing innovative teleophthalmology devices and programs.

Finally, developing an emergency contingency plan for DR management is essential to provide uninterrupted care during crises. Clinicians bear a moral and ethical responsibility to devise strategies to minimize treatment interruptions. Key elements of such a plan include maintaining a comprehensive database of DR patients and their demographics for guided medication distribution and personnel deployment, implementing backup power sources for electricity outages, ensuring access to electronic medical records, and refrigeration for temperature-sensitive medications. Adequate medication and supply reserves ahead of disasters and establishing post-disaster patient communication methods are also vital.

It is essential to acknowledge the limitations of this study, including the absence of specific data on the intersection of DR and natural disasters, as well as the lack of tailored recommendations for the unique circumstances in Puerto Rico. However, this critical analysis aims to provide guidance to ophthalmologists in Puerto Rico for effectively managing DR patients during responses to hurricanes, earthquakes, and potential future pandemics like COVID-19. The findings should inspire collaborative efforts among scientists and stakeholders in Puerto Rico to address barriers hindering patient access to teleophthalmology services, especially during times of crisis.

## Conclusions

In conclusion, given the heightened risks posed by pandemics and natural disasters, developing a robust emergency plan for managing DR is imperative. This urgency is underscored by the heightened vulnerability of regions like Puerto Rico, where the prevalence of diabetes and its complications is substantial. An effective plan should encompass established emergency measures and embrace emerging technological advancements within the field of ophthalmology.

## Appendices

Appendix A: search term combination list

1. Diabetic retinopathy AND disaster preparedness

2. Diabetic retinopathy AND disaster preparedness AND intravitreal injections

3. Diabetic retinopathy AND disaster preparedness AND anti-vascular endothelial growth factor

4. Diabetic retinopathy AND disaster preparedness AND anti-VEGF

5. Diabetic retinopathy AND emergency preparedness

6. Diabetic retinopathy AND natural disasters

7. Diabetic retinopathy AND hurricanes

8. Diabetic retinopathy AND Hurricane Maria

9. Diabetic retinopathy AND Hurricane Maria AND prevalence

10. Diabetic retinopathy AND Hurricane Irma

11. Diabetic retinopathy AND Hurricane Irma AND prevalence

12. Diabetic retinopathy AND earthquakes

13. Diabetic retinopathy AND pandemic

14. Diabetic retinopathy AND COVID-19

15. Diabetic retinopathy AND COVID

16. Diabetic retinopathy AND coronavirus

17. Diabetic retinopathy AND COVID-19 AND Puerto Rico

18. Diabetic retinopathy AND COVID AND Puerto Rico

19. Diabetic retinopathy AND coronavirus AND Puerto Rico

20. Diabetes AND disaster preparedness

21. Diabetes AND disaster preparedness

22. Retinopathy AND prevalence AND Puerto Rico

23. Retinopathy AND intravitreal injections 

24. Retinopathy AND disaster preparedness

25. Retina AND intravitreal injections AND diabetic eye disease

26. Diabetes mellitus AND disaster preparedness

27. Puerto Rico AND natural disasters
